# Rational Design of Cyclic Antimicrobial Peptides Based on BPC194 and BPC198

**DOI:** 10.3390/molecules22071054

**Published:** 2017-06-24

**Authors:** Anna D. Cirac, Maria Torné, Esther Badosa, Emilio Montesinos, Pedro Salvador, Lidia Feliu, Marta Planas

**Affiliations:** 1 LIPPSO, Departament de Química, University of Girona, Maria Aurèlia Capmany 69, 17003 Girona, Spain; anna.dcirac@gmail.com (A.D.C.); u1921061@campus.udg.edu (M.T.); 2 Institut de Química Computacional i Catàlisi i Departament de Química, University of Girona, Maria Aurèlia Capmany 69, 17003 Girona, Spain; 3 Laboratory of Plant Pathology, Institute of Food and Agricultural Technology-CIDSAV-XaRTA, University of Girona, Maria Aurèlia Capmany 61, 17003 Girona, Spain; esther.badosa@udg.edu (E.B.); emilio.montesinos@udg.edu (E.M.)

**Keywords:** antimicrobial peptides, rational design, molecular dynamics, cyclopeptides, plant pathogens, secondary structure

## Abstract

A strategy for the design of antimicrobial cyclic peptides derived from the lead compounds c(KKLKKFKKLQ) (**BPC194**) and c(KLKKKFKKLQ) (**BPC198**) is reported. First, the secondary β-structure of **BPC194** and **BPC198** was analyzed by carrying out molecular dynamics (MD) simulations. Then, based on the sequence pattern and the β-structure of **BPC194** or **BPC198**, fifteen analogues were designed and synthesized on solid-phase. The best peptides (**BPC490**, **BPC918,** and **BPC924**) showed minimum inhibitory concentration (MIC) values <6.2 μM against *Pseudomonas syringae* pv. *syringae* and *Xanthomonas axonopodis* pv. *vesicatoria*, and an MIC value of 12.5 to 25 μM against *Erwinia amylovora*, being as active as **BPC194** and **BPC198**. Interestingly, these three analogues followed the structural pattern defined from the MD simulations of the parent peptides. Thus, **BPC490** maintained the parallel alignment of the hydrophilic pairs K^1^–K^8^, K^2^–K^7^, and K^4^–K^5^, whereas **BPC918** and **BPC924** included the two hydrophilic interactions K^3^–Q^10^ and K^5^–K^8^. In short, MD simulations have proved to be very useful for ascertaining the structural features of cyclic peptides that are crucial for their biological activity. Such approaches could be further employed for the development of new antibacterial cyclic peptides.

## 1. Introduction

The treatment of plant diseases is a paramount issue in agriculture due to the economic losses caused by bacteria and fungi [1,2]. The main agents used in crop protection, copper derivatives and antibiotics, are environmental contaminants and plant pathogens may develop resistance to them [3,4]. Therefore, the discovery of new compounds that are effective against plant pathogens is essential to meet the challenges of traditional treatments.

Antimicrobial peptides (AMPs) possess favorable properties that make them promising candidates to fulfil the need of useful agents in plant protection [5,6,7,8]. They are short, cationic sequences that contain up to 50% of hydrophobic residues and are able to adopt an amphipathic structure. These peptides display a broad spectrum of activity, selectivity towards microbial targets, and a low frequency in developing microbial resistance [9,10,11,12,13,14,15,16]. Their unique mechanism of action is the basis for these properties. After an initial electrostatic interaction with the anionic phospholipids of the microbial cell membrane, AMPs have been reported to compromise the membrane bilayer via a carpet, barrel stave, toroidal pore or a disordered toroidal pore model. Furthermore, it has been shown that they can interact with intracellular targets [17,18].

The goal in the development of AMPs is to optimize their structural properties in order to enhance their antimicrobial activity. The rational design of AMPs maintaining the crucial features of native antibacterial peptides has led to the development of compounds with remarkable activity. For instance, synthetic cationic peptides V1 to V7 [19] were designed to be structurally similar to cyclic β-sheet peptides, such as protegrin 1, thanatin, and androctonin [20]. To enhance the antimicrobial activity, the common features of AMPs were modulated strategically while preserving the size, symmetry, and amphypathic structure. A similar avenue was pursued by Lee and coworkers [21]. In their study, the cyclic peptide gramicidin S was taken as a reference structure for the design of new biologically active peptides. After a systematic analysis of the effect of the ring size and the alteration of the amphipathicity and hydrophobicity, the authors were able to design a peptide (GS14K4) with a high degree of specificity for microbial cells.

On the other hand, computational tools and, in particular, molecular dynamics (MD) simulations, can provide valuable information on the membrane interaction of AMPs and their subsequent activity. Since the pioneer works of Egberts [22] and Wendoloski [23], significant efforts have been invested in achieving accurate representations of lipid bilayers and lipid–peptide interactions, that have ultimately permitted the elucidation of the mechanism of action of AMPs with atomic resolution [24,25,26,27,28,29,30].

In previous studies, we aimed to find suitable agents to control plant diseases caused by the bacteria *Xanthomonas axonopodis* pv. *vesicatoria*, *Pseudomonas syringae* pv. *syringae*, and *Erwinia amylovora,* which are responsible for important economic losses. In particular, *X. axonopodis* pv. *vesicatoria* causes the bacterial spot of tomato and pepper, *P. syringae* pv. *syringae* is the causal agent of several blight diseases, and *E. amylovora* causes the fire blight of rosaceous plants [1,2]. In this context, we designed and synthesized a library of 66 cyclic decapeptides with a general structure of c(X_5_-Phe-X_3_-Gln), where X is Lys or Leu [31]. The screening of this library against these bacteria led to the identification of the lead peptides c(KKLKKFKKLQ) (**BPC194**) and c(KLKKKFKKLQ) (**BPC198**) with minimum inhibitory concentration (MIC) values between 3.1 and 25 μM.

**Figure 1:**
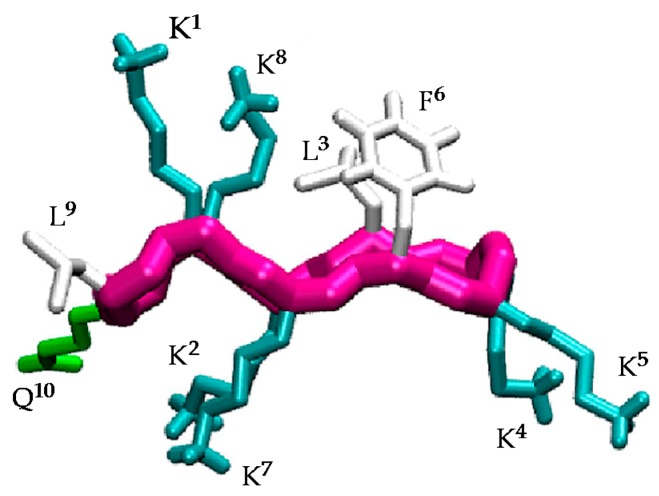
Structure of the active conformation of **BPC194** showing the side-chain alignments.

Understanding the structural factors that govern the antimicrobial activity of a library of cyclic peptides is a daunting task. In previous works [30,31,32], a combination of MD simulations and biophysical experiments has highlighted the importance of the specific arrangement of the residues in **BPC194** for the formation of stable pores and for inducing the fusion of lipid membranes. In these simulations, a stable β-structure and a symmetric arrangement of the Lys side chains was observed for the peptides inserted in the pore (Figure 1). In particular, the polar side chains of residues K^1^–K^8^, K^2^–K^7^, and K^4^–K^5^ aligned, resulting in a structure reminiscent of an anchor that helped to stabilize the polar head groups of the lipids and the water channel. Based on this, we believe that this structural motif plays a fundamental role in the antimicrobial activity of this peptide and that the precise orientation and alignment of the side chains is essential for its function.

Thus, the focus of this work was to report a strategy for the design of new antimicrobial cyclic peptides. We first analysed the primary structure of a selected number of cyclic peptides of our library to find the structural features that might govern their activity. Then, we performed MD simulations to check the stability and vulnerability of the secondary β-structure of **BPC194** and **BPC198**, with the aim of identifying the factors behind the formation of this structure. Finally, new cyclic peptides were rationally designed following a series of criteria that might help to establish the governing factors for antimicrobial activity.

## 2. Results and Discussion

### 2.1. Structural Factors Governing the Activity of Cyclic Peptides BPC194 and BPC198

The β-structure of **BPC194** induces the alignment of the polar side-chains of residues K^1^–K^8^, K^2^–K^7^, and K^4^–K^5^, as well as a spatial alignment of the side chains of the hydrophobic residues L^3^–F^6^ (Figure 1) [32]. As previously mentioned, this spatial orientation plays an important role in the antimicrobial activity of **BPC194**. The aligned Lys residues allow the formation of a stable water channel which has been established as the leading mechanism of action of this peptide [32]. Therefore, the rational design of new candidates should be based on a primary structure compatible with the formation of these key motifs.

On the other hand, **BPC198** differs from **BPC194** in terms of the residues at positions 2 and 3, precluding the alignment of residues K^2^–K^7^ and L^3^–F^6^, and yet its antimicrobial character is essentially retained [31]. So, it is clear that the primary structure for **BPC198** does not follow the same structure–function model induced for **BPC194**. Thus, it is likely that **BPC198** might exhibit an alternative folding that could permit other polar and hydrophobic alignments to take place, whilst maintaining its antimicrobial activity.

In order to validate these assumptions, we undertook MD simulations of both **BPC194** and **BPC198** peptides in a water environment. In the former case, the aim was to confirm that the active secondary structure in the membrane is also stable in water. It is worth noting that in our previous MD simulations, **BPC194** remained in an unfolded state for the first 50 ns, just before the formation of a stable β-structure upon binding to a lipid membrane [30,32].

On the contrary, in the case of **BCP198**, the aim was to prove that the structure–function model derived from **BPC194** is not suitable for this active peptide. It was hoped that the result of these MD simulations would provide an alternative model from which additional new analogues could be rationally designed and tested.

### 2.2. Stable Secondary Structure of BPC194 and BPC198

**Figure 2:**
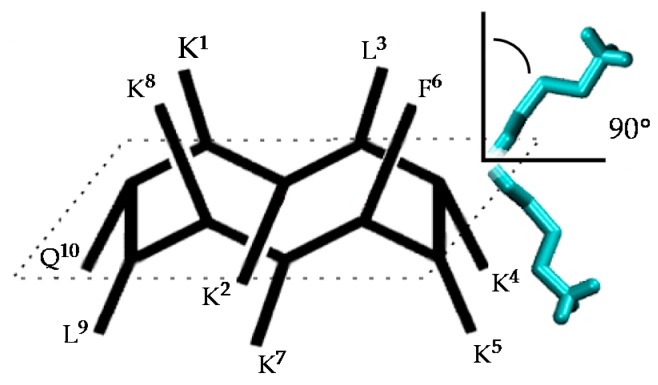
Structure of the active conformation of **BPC194** and representation of the angle formed between the normal vector to the backbone plane and a vector representing a side chain.

All MD simulations were performed for systems containing a single cyclic peptide unit solvated by water. The secondary structure of **BPC194** was taken as a reference. The starting peptide structure was characterized by a β-sheet conformation where the polar and hydrophobic side chains were aligned (Figure 2). All systems were simulated in a cubic box of length ca. 4 nm and about 1600–2100 water molecules, with Cl^−^ to neutralize the system.

The spatial arrangement of the side-chain pairs and the stability of the β-structure formation were analyzed along the trajectories. The time evolution of the secondary structure of the peptides was determined with the Dictionary of Secondary Structures of Proteins (DSSP) protocol and through the monitoring of hydrogen bond distances in the backbone. The angle between the normal vector to the (approximate) plane formed by the backbone atoms and the vector representing the side chains was used to represent the spatial arrangement of the side chains, and as a measure of amphipathicity. As shown in Figure 2, small values of this angle (*<*90°) indicate that the corresponding side chain is arranged on the upper face of the peptide, whereas values larger than 90° correspond to the bottom face.

**Figure 3:**
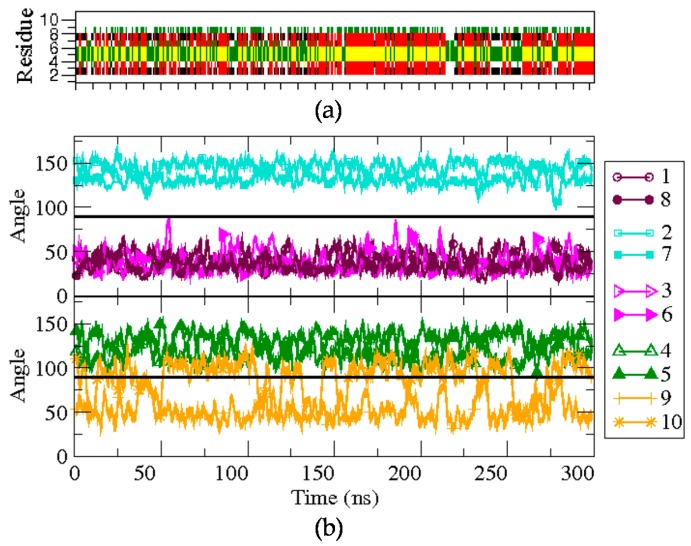
Structural characterization of the **BPC194** simulation. (**a**) The secondary structure as a function of time is calculated by DSSP. The colors denote the β-sheet (red), the turn (yellow), β-bridge (black), bend (green), and random coil elements (white/empty). (**b**) Time evolution of the angle formed between the normal vector to the backbone plane and a vector representing each of the side chains.

Firstly, **BPC194** in its folded state was simulated for over 300 ns in solution. The structural features of the trajectory are depicted in Figure 3 (bottom panel). The side chains of the pair K^2^–K^7^ are located at the lower face of the peptide backbone during the whole simulation (>90°), with little angle fluctuations (cyan curves). Similarly, the residues of the pairs K^1^–K^8^ and L^3^–F^6^ also appear to be aligned, but placed on the upper face of the peptide (<90°). The hydrophilic residues suffer somewhat fewer fluctuations compared to the hydrophobic pair due to the formation of inter-chain hydrogen bonds. The side chains of the remaining pair of residues, K^4^–K^5^ and L^9^–Q^10^, which are part of the turn regions, show a more flexible behavior. While the values of the angles for the pair K^4^–K^5^ often oscillate near 100°, quite close to co-planarity with the backbone, the side chains of residues L^9^ and Q^10^ exhibit larger fluctuations and oscillate between the upper and lower face during the whole simulation (orange curves). Despite fluctuations, the overall location of the side-chains gives rise to an amphipathic structure that is stable during the whole simulation.

The DSSP plot (upper panel of Figure 3) shows that the secondary structure in solution is quite stable when starting from the folded state. Indeed, the starting β-structure persists around 39% of the simulation, albeit with minor fluctuations. The hydrogen bond distance between the backbone’s oxygen donor atom of residues K^8^ or F^6^ and the hydrogen acceptor of K^1^ or L^3^, respectively, was also monitored. The hydrogen bonds between these residues were maintained during most of the simulation time, with average distances of 0.22 ± 0.07 nm (K^1^–K^8^) and 0.28 ± 0.06 nm (L^3^–F^6^).

As mentioned above, in previously reported MD simulations of **BPC194** [30,32], the peptide was essentially in an unfolded state in a water environment for ca 50 ns, prior to forming a β-structure upon binding to lipid membranes. The long simulations in a water environment described here show a seemingly opposite view, where starting from a folded state, the structure remains quite stable for as long as 300 ns. So the lipid membrane and possibly the presence of other peptides induce the formation of a stable specific active structure that is otherwise more difficult to form in solution. Nevertheless, the fact that the active structure is stable in water opens up the possibility of studying other peptides of the library in similar conformations to that of **BPC194** in the same conditions.

**Figure 4:**
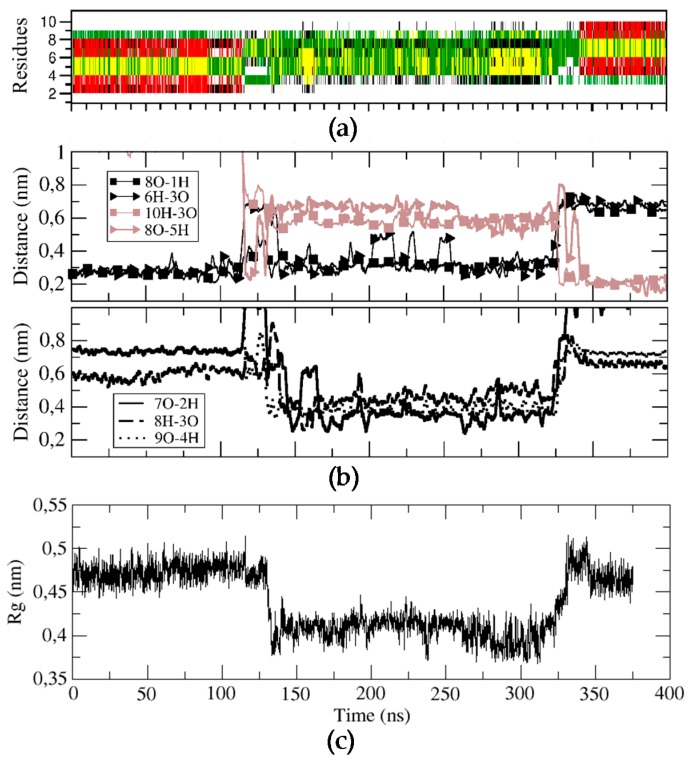
Structural characterization of the **BPC198** simulation. (**a**) DSSP plot, (**b**) time evolution of the relevant hydrogen bond distances and (**c**) radius of gyration (nm) of the backbone structure along the trajectory.

For **BPC198**, we carried out an analogous simulation starting from the same secondary structure as for **BPC194**, but replacing the residues at positions 2 and 3 with a Leu and a Lys, respectively. This initial structure was first modeled in silico, followed by energy minimization steps to remove the bad contacts. The qualitative analysis of the possible alignment of the side chains of the amino acids already indicated a mismatch of polar and hydrophobic residues (L^2^–K^7^, K^3^–F^6^), pointing out that the structure might not be stable (Figure 4c). This fact suggested that **BPC198** might exhibit alternative folding with other polar and hydrophobic alignments. This is indeed the case and **BPC198** showed a very different behavior in the water phase. As shown in Figure 4a, the peptide unfolded after around 100 ns and the loss of the secondary structure was not immediately followed by an alternative rearrangement of residues to a stable conformation up until 340 ns. During that time lapse, the peptide adopted an unstructured conformation, only presenting turns due to the cyclic nature of the molecule.

To characterize this unfolding/folding process, several key backbone hydrogen bonds were also monitored during the simulation. The initial hydrogen bond interactions between residues K^1^–K^8^ and K^3^–F^6^ were stable till around 120 ns (Figure 4b). At this point, a sudden fluctuation led to a simultaneous breaking of both hydrogen bonds and a concomitant approach of residues K^5^–K^8^ and L^3^–Q^10^ to hydrogen bonding distances. Moreover, the structure became more compact, as indicated by the decrease in the radius of gyration of the cyclic peptide (Figure 4, bottom panel) and by the shorter distances observed between residues K^7^–L^2^, K^8^–K^3^, and L^9^–K^4^, which even led to the temporary formation of transient weak hydrogen bonds (Figure 4, third panel). In addition, after around 150 ns, the distances of the initial hydrogen bonds between K^1^–K^8^ and K^3^–F^6^ slightly increased and oscillated until approximately 340 ns, at which point a large fluctuation occurred leading to a new stable β-structure. This conformation was characterized by strong and stable hydrogen bonds between residues K^5^–K^8^ and L^3^–Q^10^ (Figure 4, second panel, brown curves).

The role of weak transient interactions on the transition between folded states has already been discussed in the literature. For instance, the folding of a charged 21-amino acid peptide (con-T) showed a multi-step folding pathway, where hydrophobic interactions were not the major driving force of the folding process. Instead, charged–charged interactions and salt-bridge interactions amongst the peptide structure produced different local peptide shapes that, after consecutively broken-formation interactions, led the peptide to its folded state [33].

**Figure 5:**
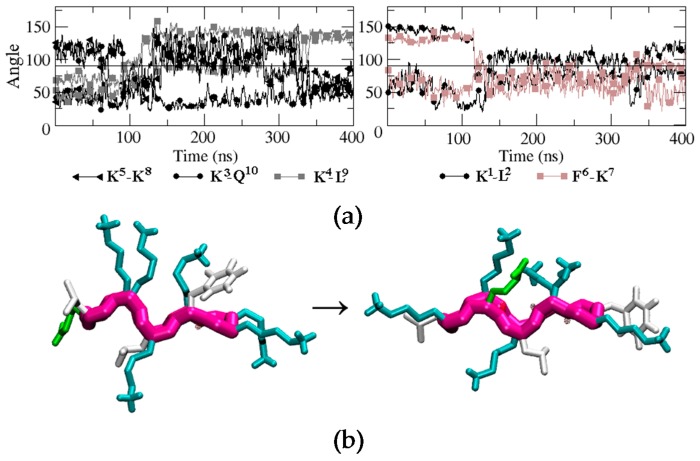
(**a**) Time evolution of the angle formed between the normal vector to the backbone plane and the vector representing each of the side chains. Black, brown, and grey curves correspond to hydrophilic, hydrophobic, and hydrophobic-hydrophilic pair residues, respectively. (**b**) Snapshots of **BPC198** at 0 ns (left) and at 350 ns (right).

To evaluate how the side chains arrange in space during the unfolding/folding process and whether they align in the upper or in the lower face of the peptide, we analyzed the time evolution of the angles similarly to **BPC194**. The analysis is displayed in Figure 5. One can observe that the initial structure remained quite constant until around 80 ns. At this point, the side chain of residue Q^10^ suddenly migrated from the lower face of the peptide to the upper face and temporarily aligned with the K^3^ side-chain, which remained in the upper face during the whole simulation. A similar process occurred at the same time for the side chain of residue K^5^. In this case, however, after aligning with the side chain of K^8^, both started to oscillate around an angle of 90° until the final structure was formed at around 325 ns. These events occurred at a shorter time (80 ns) than the breaking of the intramolecular hydrogen bonds in the backbone (120 ns), suggesting that the latter process was induced by the oscillation of the side chains and not the opposite. In the case of residues K^1^, L^2^, F^6^, and K^7^, their side chains started to oscillate after 120 ns around a parallel position with respect to the backbone (angle close to 90°). Finally, after 325 ns, just before the formation of the backbone hydrogen bonds that stabilize the final β-structure (340 ns), the side-chain of residue Q^10^ swung again to the upper face of the peptide and aligned with K^3^ until the end of the simulation. The residue pair K^5^–K^8^ also aligned in the upper face of the conformation, but the angle (around 60°) was larger than that expected for the interaction of two polar residues. The side chains of K^1^, L^2^, F^6^, and K^7^ ended up in the turn regions of the final β-structure and did not align.

A representative snapshot of the final β-structure of **BPC198** is depicted at the bottom of Figure 5. The conformation is reminiscent of the active structure of **BPC194** where two pairs of polar side chains are spatially aligned and arranged in one side of the molecule. However, in this case, the side chains of a Lys (polar) and a Leu (hydrophobic) are forced to align in the lower face due to the presence of tight hydrogen bonds in the backbone. So, this structure is not as amphipathic as the one observed for **BPC194**. To ensure that the new structure was indeed stable in solution, an additional MD simulation was performed starting from the new β-structure, which remained stable along the time course of the simulation (400 ns).

### 2.3. Design and Synthesis of **BPC194** and **BPC198** Analogues

With the information gathered from the experiments and simulations described above, we designed new **BPC194** and **BPC198** analogues to validate our structure–function model and to identify cyclic peptides with antimicrobial activity.

**Figure 6:**
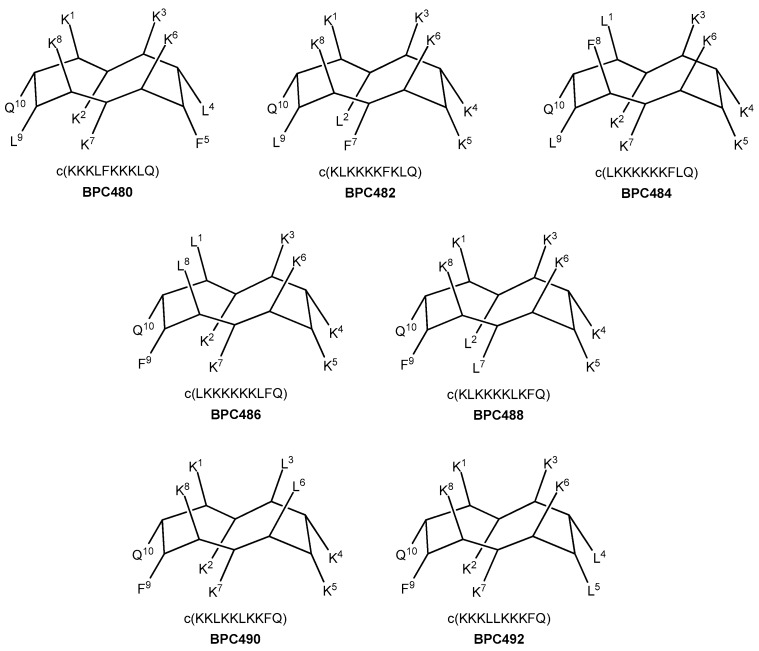
Sequence and hypothetic structure of the **BPC194** analogues.

Seven analogues were designed, taking as reference the sequence pattern and active secondary structure of **BPC194**. The number of hydrophobic and hydrophilic residues, i.e., six Lys, two Leu, one Phe, and one Gln, was maintained, as well as the parallel alignment of the three hydrophilic K–K pairs. Thus, in three analogues (four including **BPC194**), the hydrophobic residues involved Q–L and L–F pairs (analogues **BPC480**, **BPC482**, and **BPC484**), whereas in the other four analogues, the pairing would take place between the Q–F and L–L residues (analogues **BPC486**, **BPC488**, **BPC490**, and **BPC492**). The primary structure and the hypothetic conformation of the seven **BPC194** analogues are depicted in Figure 6. Peptides differed in terms of the relative distribution of the hydrophilic K–K and hydrophobic pairs on the upper and lower regions of the hypothetic active structure.

**Figure 7:**
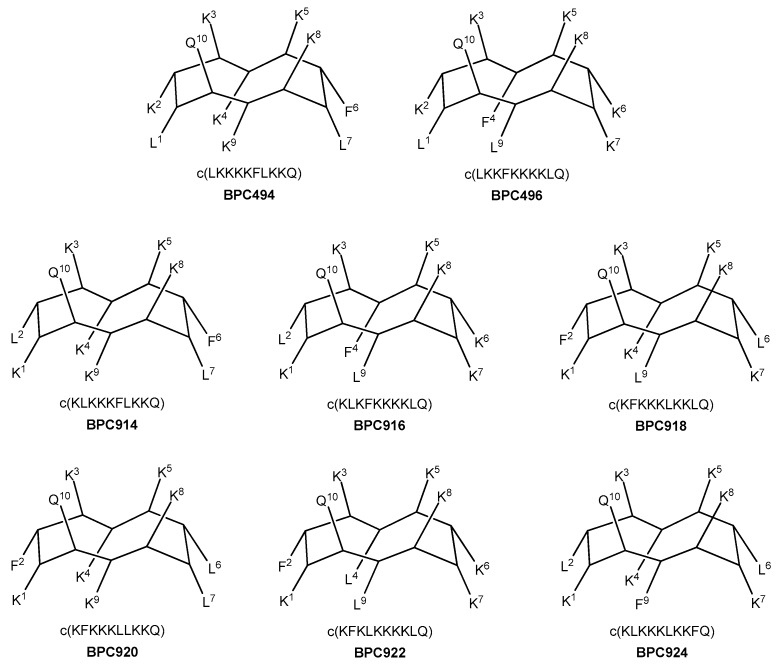
Sequence and hypothetic structure of the **BPC198** analogues.

Additional analogues were designed, taking as a reference the new stable β-structure obtained for **BPC198** (Figure 7). Six out of the eight analogues designed maintained the K^3^–Q^10^ and K^5^–K^8^ pairs in the upper face of the molecule (**BPC494**, **BPC496**, **BPC914**, **BPC916**, **BPC920**, and **BPC922**), and differed from **BPC198** in the formation of an additional hydrophilic K–K pair, a motif repeatedly observed in this work. The other two analogues, **BPC918** and **BPC924**, included the two hydrophilic interactions present in **BPC198**, K^3^–Q^10^ and K^5^–K^8^, and differed only in the position of the hydrophobic residues.

**Scheme 1:**
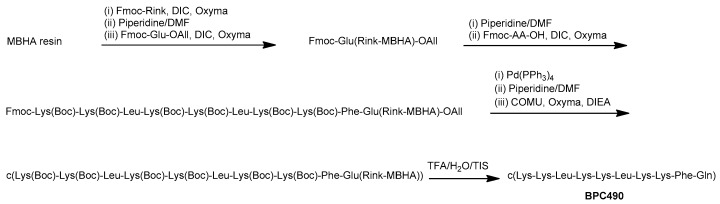
General strategy for the synthesis of the cyclic decapeptides represented for **BPC490**.

The synthesis of the cyclic decapeptides was carried out on a 4-methylbenzhydrylamine (MBHA) resin (0.4 mmols/g) following a 9-fluorenylmethoxycarbonyl (Fmoc)/*tert*-butyl (*t*-Bu)/Allyl strategy by carrying out the solid-phase synthesis of the linear sequence followed by on-resin cyclization (Scheme 1). The *tert*-butyloxycarbonyl (Boc) group was used as side-chain protection for Lys. Fmoc-Glu-OAll was attached to the solid support through the side chain to serve as a peptide anchoring point to the support, and resulted in a Gln after peptide cleavage from the resin. After the coupling of the Fmoc-Rink linker, the linear peptidyl resin was constructed by sequential Fmoc deprotection and coupling steps. Fmoc group removal was performed with piperidine/DMF (3:7) and amino acid couplings were mediated by *N,N*-diisopropylcarbodiimide (DIC) and ethyl 2-cyano-2-(hydroxyimino)acetate (Oxyma). Once the synthesis of the linear peptidyl resin was completed, the C-terminal allyl ester cleavage was achieved by treatment with Pd(PPh_3_)_4_ in CHCl_3_-AcOH-*N*-methylmorpholine (NMM) (37:2:1) and it was followed by *N*^α^-Fmoc group removal. Cyclization was then performed using 1-[(1-(cyano-2-ethoxy-2-oxoethylideneaminooxy)-dimethylamino-morpholinomethylene)] methanaminium hexafluorophosphate (COMU), Oxyma, and *N,N*-diisopropylethylamine (DIEA). Finally, the resulting cyclic decapeptides were acidolytically cleaved, analyzed by high-performance liquid chromatography (HPLC), and characterized by mass spectrometry. They were obtained in HPLC purities ranging from 94 to >99%.

### 2.4. Antibacterial Activity of the Designed Cyclic Decapeptides

The antibacterial activity of the designed cyclic peptides was assayed against *P. syringae* pv. *syringae*, *X. axonopodis* pv. *vesicatoria*, and *E. amylovora* at 3.1, 6.2, 12.5, 25, and 50 μM. The results obtained are gathered in Table 1. Peptides **BPC194** and **BPC198** were also included in these assays for comparison purposes.

All peptides were active against at least one pathogen with MIC < 25 μM. *E. amylovora* was the least sensitive bacteria to these peptides. This different susceptibility is in agreement with previous reports on this family of cyclic peptides [31,34]. The different membrane composition of the target pathogen would result in a different binding rate of the peptides. Regarding the **BPC194** analogues, **BPC482**, **BPC488**, and **BPC490** displayed significant activity against *P. syringae* pv. *syringae* and *X. axonopodis* pv. *vesicatoria* with MIC < 12.5 μM. Among them, **BPC490** had an MIC value of 3.1 to 6.2 μM against these two bacteria and an MIC value of 12.5 to 25 μM against *E. amylovora*. This compound was as active as the parent peptide **BPC194**.

In the case of the eight sequences derived from **BPC198**, four of them also showed MIC < 12.5 μM against *P. syringae* pv. *syringae* and *X. axonopodis* pv. *vesicatoria*. Notably, the two sequences **BPC918** and **BPC924** were as active as **BPC198** against *P. syringae* pv. *syringae* (MIC of 3.1 to 6.2 μM) and *E. amylovora* (MIC of 12.5 to 25 μM), and more active against *X. axonopodis* pv. *vesicatoria* (MIC of 1.6 to 3.1 μM vs. 3.1 to 6.2 μM).

Noticeably, the analysis of these results reveals that the **BPC194** analogue that displays the best biological activity profile, **BPC490**, is the only one that incorporates the three hydrophilic pairs K^1^–K^8^, K^2^–K^7^, and K^4^–K^5^, differing from **BPC194** in only the composition of the hydrophobic pairs. Thus, **BPC490** is able to adopt the same spatial disposition of the hydrophilic and hydrophobic pairs as **BPC194**.

**Figure 8:**
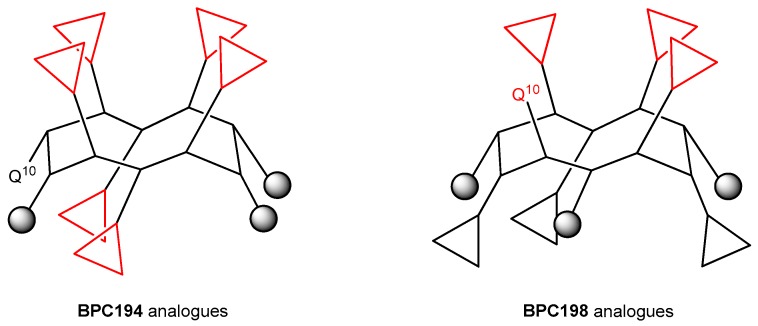
General structure of the active conformation of **BPC194** and **BPC198** analogues. Triangles stand for hydrophilic Lys residues and grey circles stand for hydrophobic Leu or Phe residues.

The results from the **BPC198** analogues demonstrate that the presence of three hydrophilic K–K pairs leads to a decrease in the antibacterial activity. In fact, the two best analogues, **BPC918** and **BPC924**, contain the two hydrophilic pairs K^3^–Q^10^ and K^5^–K^8^ present in **BPC198** and differ from the parent peptide in the hydrophobic residues at positions 2, 6, and 9 (Leu or Phe). These observations confirm the hypothesis of the structure-function model inferred from our MD simulations for **BPC194** and **BPC198**. The general structure of the active conformation of these two families of cyclic peptides is depicted in Figure 8.

## 3. Materials and Methods

### 3.1. Chemicals and Instruments

Commercially available reagents were used throughout, without purification. Solvents were purified and dried by passing them through an activated alumina purification system (MBraun SPS-800, MBraun, Garching, Germany) or by conventional distillation techniques.

All compounds were analyzed under standard analytical HPLC conditions with a Dionex liquid chromatography instrument (Dionex, Germering, Germany). Detection was performed at 220 nm. Analysis was carried out using the Dionex instrument with a Kromasil 100 C_18_ (40 mm × 4.6 mm, 3.5 μm, Agilent Technologies, Barcelona, Spain) column with a 2%–100% B linear gradient over 17 min at a flow rate of 1 mL/min. Solvent A was 0.1% aqueous trifluoroacetic acid (TFA) and solvent B was 0.1% TFA in CH_3_CN.

Electrospray ionization (ESI) mass spectrometry (MS) analyses were performed with an Esquire 6000 ESI ion Trap LC/MS (Bruker Daltonics, Madrid, Spain) instrument equipped with an electrospray ion source (Serveis Tècnics de Recerca of the University of Girona). The instrument was operated in the positive ESI(+) ion mode. Samples (5 μL) were introduced into the mass spectrometer ion source directly through an HPLC autosampler (Agilent Technologies). The mobile phase (80:20 CH_3_CN/H_2_O at a flow rate of 100 μL/min) was delivered by a 1100 Series HPLC pump (Agilent Technologies). Nitrogen was employed as both the drying and nebulizing gas.

High resolution mass spectrometry (HRMS) spectra were recorded under conditions of ESI with a Bruker MicrOTOF-Q IITM instrument (Bruker, Serveis Tècnics de Recerca of the University of Girona) using a hybrid quadrupole time-of-flight mass spectrometer. Samples were introduced into the mass spectrometer ion source by direct infusion through a syringe pump (Bruker, University of Girona) and were externally calibrated using sodium formate. The instrument was operated in the positive ESI(+) ion mode.

### 3.2. Synthesis of the Cyclic Peptides

The cyclic peptides were synthesized manually by the solid-phase method using standard Fmoc chemistry on an MBHA resin (0.4 mmol/g). Fmoc-Glu-OAll, Fmoc-Leu-OH, Fmoc-Phe-OH, and Fmoc-Lys(Boc)-OH were used as amino acid derivatives. The resin was swollen with CH_2_Cl_2_ (1 × 20 min) and DMF (1 × 20 min), and washed with piperidine/DMF (3:7, 1 × 5 min), DMF (6 × 1 min), and CH_2_Cl_2_ (3 × 1 min). Then, the Fmoc-Rink linker (5 eq) was coupled to the resin in the presence of Oxyma (4 eq) and DIC (4 eq) in *N,N*-dimethylformamide (DMF) for 2 h. After this time, the resin was washed with DMF (6 × 1 min) and CH_2_Cl_2_ (3 × 1 min). The linear sequences were prepared by sequential steps of Fmoc group removal, amino acid couplings, and washings. The Fmoc group was removed with piperidine/DMF (3:7, 5 × 10 min). Couplings of the corresponding Fmoc-protected amino acid (4 eq) were mediated by Oxyma (4 eq) and DIC (4 eq) in DMF for 2 h. The completion of the reactions was checked with the Kaiser test [35]. After each coupling and deprotection step, the resin was washed with DMF (6 × 1 min) and CH_2_Cl_2_ (3 × 1 min). After the fifth amino acid coupling, *N*-methyl-2-pyrrolidinone (NMP) was used as the solvent. Once the peptidyl chain was completed, the C-terminal allyl ester was cleaved by treatment with Pd(PPh_3_)_4_ (3 eq) in CHCl_3_/AcOH/NMM (37:2:1) under nitrogen for 3 h. The resin was then washed with tetrahydrofuran (THF) (3 × 2 min), NMP (3 × 2 min), CH_2_Cl_2_ (3 × 2 min), DIEA/ CH_2_Cl_2_ (1:19, 3 × 2 min), sodium *N,N*-diethyldithiocarbamate (0.03 M in NMP, 3 × 15 min), NMP (10 × 1 min), CH_2_Cl_2_ (3 × 2 min), and NMP (3 × 2 min). Fmoc was removed with piperidine/NMP (3:7, 2 + 10 min), followed by washes with NMP (6 × 1 min) and CH_2_Cl_2_ (3 × 1 min). Cyclization was carried out by treating the resin with Oxyma (5 eq), COMU (5 eq), and DIEA (10 eq) in NMP at 25 °C for 24 h. The completion of the reaction was checked with the Kaiser test [35]. Following washes with NMP (6 × 1 min) and CH_2_Cl_2_ (3 × 1 min), cyclic decapeptides were cleaved from the resin by treatment with TFA/H_2_O/triisopropylsilane (TIS) (95:2.5:2.5) for 1 h. The cleavage cocktail was then evaporated and, after diethyl ether extractions, the cyclic peptides were dissolved in H_2_O, lyophilized, analyzed by reverse-phase (RP)-HPLC, and characterized by mass spectrometry.

**BPC480**. Retention time, *t*_R_ = 5.81 min (96% purity). MS (ESI): *m*/*z* = 1271.0 [M + H]^+^, 1293.0 [M + Na]^+^. HRMS (ESI): calcd. for C_62_H_113_N_17_O_11_ [M + 2H]^2+^ 635.9397, found 635.9424; calcd. for C_62_H_112_N_17_O_11_ [M + H]^+^ 1270.8722, found 1270.8723.

**BPC482**. *t*_R_ = 5.96 min (98% purity). MS (ESI): *m*/*z* = 1271.0 [M + H]^+^, 1293.0 [M + Na]^+^. HRMS (ESI): calcd. for C_62_H_113_N_17_O_11_ [M + 2H]^2+^ 635.9397, found 635.9416; calcd. for C_62_H_112_N_17_O_11_ [M + H]^+^ 1270.8722, found 1270.8700.

**BPC484**. *t*_R_ = 5.95 min (97% purity). MS (ESI): *m*/*z* = 1271.0 [M + H]^+^, 1293.0 [M + Na]^+^. HRMS (ESI): calcd. for C_62_H_113_N_17_O_11_ [M + 2H]^2+^ 635.9397, found 635.9411; calcd. for C_62_H_112_N_17_O_11_ [M + H]^+^ 1270.8722, found 1270.8694.

**BPC486**. *t*_R_ = 5.98 min (96% purity). MS (ESI): *m*/*z* = 1271.0 [M + H]^+^, 1293.0 [M + Na]^+^. HRMS (ESI): calcd. for C_62_H_113_N_17_O_11_ [M + 2H]^2+^ 635.9397, found 635.9427; calcd. for C_62_H_112_N_17_O_11_ [M + H]^+^ 1270.8722, found 1270.8713.

**BPC488**. *t*_R_ = 5.92 min (96% purity). MS (ESI): *m*/*z* = 1271.0 [M + H]^+^, 1293.0 [M + Na]^+^. HRMS (ESI): calcd. for C_62_H_113_N_17_O_11_ [M + 2H]^2+^ 635.9397, found 635.9426; calcd. for C_62_H_112_N_17_O_11_ [M + H]^+^ 1270.8722, found 1270.8721.

**BPC490**. *t*_R_ = 6.34 min (95% purity). MS (ESI): *m*/*z* = 1271.0 [M + H]^+^, 1293.0 [M + Na]^+^. HRMS (ESI): calcd. for C_62_H_113_N_17_O_11_ [M + 2H]^2+^ 635.9397, found 635.9420; calcd. for C_62_H_112_N_17_O_11_ [M + H]^+^ 1270.8722, found 1270.8706.

**BPC492**. *t*_R_ = 5.77 min (97% purity). MS (ESI): *m*/*z* = 1271.0 [M + H]^+^, 1293.0 [M + Na]^+^. HRMS (ESI): calcd. for C_62_H_113_N_17_O_11_ [M + 2H]^2+^ 635.9397, found 635.9426; calcd. for C_62_H_112_N_17_O_11_ [M + H]^+^ 1270.8722, found 1270.8738.

**BPC494**. *t*_R_ = 5.80 min (94% purity). MS (ESI): *m*/*z* = 1271.0 [M + H]^+^, 1293.0 [M + Na]^+^. HRMS (ESI): calcd. for C_62_H_113_N_17_O_11_ [M + 2H]^2+^ 635.9397, found 635.9414; calcd. for C_62_H_112_N_17_O_11_ [M + H]^+^ 1270.8722, found 1270.8723.

**BPC496**. *t*_R_ = 5.93 min (97% purity). MS (ESI): *m*/*z* = 1271.0 [M + H]^+^, 1293.0 [M + Na]^+^. HRMS (ESI): calcd. for C_62_H_113_N_17_O_11_ [M + 2H]^2+^ 635.9397, found 635.9427; calcd. for C_62_H_112_N_17_O_11_ [M + H]^+^ 1270.8722, found 1270.8733.

**BPC914**. *t*_R_ = 5.79 min (>99% purity). MS (ESI): *m*/*z* = 1270.9 [M + H]^+^, 1292.9 [M + Na]^+^. HRMS (ESI): calcd. for C_62_H_113_N_17_O_11_ [M + 2H]^2+^ 635.9397, found 635.9400; calcd. for C_62_H_112_N_17_O_11_ [M + H]^+^ 1270.8722, found 1270.8708.

**BPC916**. *t*_R_ = 5.88 min (>99% purity). MS (ESI): *m*/*z* = 1270.9 [M + H]^+^, 1292.9 [M + Na]^+^. HRMS (ESI): calcd. for C_62_H_113_N_17_O_11_ [M + 2H]^2+^ 635.9397, found 635.9420; calcd. for C_62_H_112_N_17_O_11_ [M + H]^+^ 1270.8722, found 1270.8733.

**BPC918**. *t*_R_ = 6.13 min (96% purity). MS (ESI): *m*/*z* = 1270.9 [M + H]^+^, 1292.9 [M + Na]^+^. HRMS (ESI): calcd. for C_62_H_113_N_17_O_11_ [M + 2H]^2+^ 635.9397, found 635.9401; calcd. for C_62_H_112_N_17_O_11_ [M + H]^+^ 1270.8722, found 1270.8701.

**BPC920**. *t*_R_ = 5.91 min (>99% purity). MS (ESI): *m*/*z* = 1292.9 [M + Na]^+^. HRMS (ESI): calcd. for C_62_H_113_N_17_O_11_ [M + 2H]^2+^ 635.9397, found 635.9370; calcd. for C_62_H_112_N_17_O_11_ [M + H]^+^ 1270.8722, found 1270.8683.

**BPC922**. *t*_R_ = 6.03 min (99% purity). MS (ESI): *m*/*z* = 1270.9 [M + H]^+^, 1292.9 [M + Na]^+^. HRMS (ESI): calcd. for C_62_H_113_N_17_O_11_ [M + 2H]^2+^ 635.9397, found 635.9398; calcd. for C_62_H_112_N_17_O_11_ [M + H]^+^ 1270.8722, found 1270.8744.

**BPC924**. *t*_R_ = 6.38 min (95% purity). MS (ESI): *m*/*z* = 1270.9 [M + H]^+^, 1292.9 [M + Na]^+^. HRMS (ESI): calcd. for C_62_H_113_N_17_O_11_ [M + 2H]^2+^ 635.9397, found 635.9397; calcd. for C_62_H_112_N_17_O_11_ [M + H]^+^ 1270.8722, found 1270.8730.

### 3.3. Bacterial Strains and Growth Conditions

The following plant pathogenic bacterial strains were used: *Erwinia amylovora* PMV6076 (Institut National de la Recherche Agronomique, Angers, France); *Pseudomonas syringae* pv. *syringae* EPS94 (Institut de Tecnologia Agroalimentària, Universitat de Girona, Spain); and *Xanthomonas axonopodis* pv. *vesicatoria* 2133-2 (Instituto Valenciano de Investigaciones Agrarias, Valencia, Spain). All bacteria were stored in Luria Bertani (LB) broth supplemented with glycerol (20%) and maintained at –80 °C. *E. amylovora* and *P. syringae* pv. *syringae* were scrapped from LB agar after growing for 24 h and *X. axonopodis* pv. *vesicatoria* after growing for 48 h at 25 °C. The cell material was suspended in sterile water to obtain a suspension of 10^8^ CFU mL^−1^.

### 3.4. Antibacterial Activity

Lyophilized peptides were solubilized in sterile Milli-Q water to a final concentration of 1000 μM and sterilized through a 0.22-μm pore filter. For MIC assessment, dilutions of the synthetic peptides were made to obtain a final concentration of 500, 250, 125, 62.5, 31.2, and 15.6 μM. Twenty microlitres of each dilution were mixed in a microtiter plate well with 20 μL of the corresponding suspension of the bacterial indicator and 160 μL of Trypticase Soy Broth (TSB) (BioMèrieux, Craponne, France) to a total volume of 200 μL. Three replicates for each strain, peptide, and concentration were used. Positive controls contained water instead of peptide and negative controls contained peptides without bacterial suspension. Bacterial growth was automatically determined by an optical density measurement at 600 nm (Bioscreen C, Labsystem, Helsinki, Finland). Microplates were incubated at 25 °C with 20 s shaking before hourly absorbance measurements for 48 h. Each experiment was repeated twice. The MIC was taken as the lowest peptide concentration with no growth at the end of the experiment. The inhibition of growth (*I*) was calculated as a percentage of the positive control using the equation: *I* = 100 × [(*AC − AS*)/*AC*], where *AC* is the area under the curve of the control, and *AS* is the area under the curve of a given peptide concentration.

### 3.5. Molecular Dynamics Simulations

The GROMACS software package [36] was used to perform MD simulations. The GROMOS force-field 43a2 [37] was used to describe the peptide and peptide–solvent interactions. The force-fields were parametrized for use with a group-based twin range cut-off scheme (using cutoffs of 1.0/1.4 nm and a pair-list update frequency of once per 10 steps), including the particle-mesh Ewald (PME) method. The water was modeled using the SPC model [38]. A time step of 2 fs was used. Bond lengths were constrained using the LINCS algorithm [39]. The simulations were performed in the NPT ensemble using periodic boundary conditions. The temperature was weakly coupled (coupling time 0.1 ps) to T = 298 K using the Berendsen thermostat [40]. The pressure was also weakly coupled (coupling time of 1.0 ps and compressibility of 4.5 × 10^−5^), using an isotropic coupling scheme at 1 bar. The DSSP protocol and the corresponding computer program [41] were employed to analyze the time evolution of the local secondary structure.

## 4. Conclusions

MD simulations have been used as a tool to recognize the factors that drive the formation of stable β-structures with a partial amphipathic character of cyclic peptides with antimicrobial activity. The stability of the active conformation of peptide **BPC194** in the lipid membrane found previously was tested by independent MD simulations in a water solution. Analogous simulations of another active peptide of the library (**BPC198**) highlighted the key role of the parallel alignment of the side-chain of the residues. At the active conformation of **BPC194**, the amino acid sequence of **BPC198** induced the mismatch of polar and hydrophobic side-chains that eventually led to the unfolding and folding of the peptide to form an alternative compact and stable β-structure. The unfolding pathway of the starting folded structure to the new, amphipathic β-structure is described in atomistic detail. Weak transient interactions assist in the transition between folded states, as already described in the literature for other small peptides.

Based on the stable active structures of peptides **BPC194** and **BPC198**, a set of fifteen cyclic peptides were rationally designed and synthesized. Three of these new analogues exhibited similar antibacterial activity as the parent peptides. These analogues maintain the number and the spatial orientation of the hydrophilic pair interactions of **BPC194** and **BPC198**, differing only in the position of the hydrophobic residues. Therefore, these results confirm the general structure of the active conformation extracted from MD simulations for **BPC194** and **BPC198**. This structure could be the basis of the design of new cyclic decapeptides to be used as antibacterial agents.

## Figures and Tables

**Table 1:** Antibacterial activity (MIC) of the cyclic decapeptides.

Code	MIC Intervals (μM)		
	*Pss* ^a^	*Xav* ^a^	*Ea* ^a^
**BPC194**	3.1-6.2	3.1–6.2	6.2–12.5
**BPC198**	3.1–6.2	3.1–6.2	12.5–25
**BPC480**	12.5–25	12.5–25	>50
**BPC482**	6.2–12.5	6.2–12.5	>50
**BPC484**	12.5–25	25–50	>50
**BPC486**	12.5–25	>50	>50
**BPC488**	6.2–12.5	6.2–12.5	>50
**BPC490**	3.1–6.2	3.1–6.2	12.5–25
**BPC492**	12.5–25	12.5–25	>50
**BPC494**	6.2–12.5	6.2–12.5	>50
**BPC496**	6.2–12.5	6.2–12.5	>50
**BPC914**	12.5–25	12.5–25	>50
**BPC916**	6.2–12.5	12.5–25	>50
**BPC918**	3.1–6.2	1.6–3.1	12.5–25
**BPC920**	12.5–25	25–50	>50
**BPC922**	12.5–25	12.5–25	>50
**BPC924**	3.1–6.2	1.6–3.1	12.5–25

^a^ Pss stands for *Pseudomonas syringae* pv. *syringae*; Xav stands for *Xanthomonas axonopodis* pv. *vesicatoria*; Ea stands for *Erwinia amylovora*.
